# Genome-Scale Metabolic Modeling for Unraveling Molecular Mechanisms of High Threat Pathogens

**DOI:** 10.3389/fcell.2020.566702

**Published:** 2020-11-03

**Authors:** Mustafa Sertbas, Kutlu O. Ulgen

**Affiliations:** ^1^Department of Chemical Engineering, Bogazici University, Istanbul, Turkey; ^2^Department of Chemical Engineering, Istanbul Technical University, Istanbul, Turkey

**Keywords:** genome-scale metabolic models, pathogen, systems biology, pathogen-host interactions, gene essentiality, flux balance analysis (FBA), flux variability analysis, infection

## Abstract

Pathogens give rise to a wide range of diseases threatening global health and hence drawing public health agencies' attention to establish preventative and curative solutions. Genome-scale metabolic modeling is ever increasingly used tool for biomedical applications including the elucidation of antibiotic resistance, virulence, single pathogen mechanisms and pathogen-host interaction systems. With this approach, the sophisticated cellular system of metabolic reactions inside the pathogens as well as between pathogen and host cells are represented in conjunction with their corresponding genes and enzymes. Along with essential metabolic reactions, alternate pathways and fluxes are predicted by performing computational flux analyses for the growth of pathogens in a very short time. The genes or enzymes responsible for the essential metabolic reactions in pathogen growth are regarded as potential drug targets, as *a priori* guide to researchers in the pharmaceutical field. Pathogens alter the key metabolic processes in infected host, ultimately the objective of these integrative constraint-based context-specific metabolic models is to provide novel insights toward understanding the metabolic basis of the acute and chronic processes of infection, revealing cellular mechanisms of pathogenesis, identifying strain-specific biomarkers and developing new therapeutic approaches including the combination drugs. The reaction rates predicted during different time points of pathogen development enable us to predict active pathways and those that only occur during certain stages of infection, and thus point out the putative drug targets. Among others, fatty acid and lipid syntheses reactions are recent targets of new antimicrobial drugs. Genome-scale metabolic models provide an improved understanding of how intracellular pathogens utilize the existing microenvironment of the host. Here, we reviewed the current knowledge of genome-scale metabolic modeling in pathogen cells as well as pathogen host interaction systems and the promising applications in the extension of curative strategies against pathogens for global preventative healthcare.

## Introduction

Pathogens give rise to a wide range of diseases threatening global health and drawing public health agencies' attention to establish preventative and curative solutions (Sweileh, 2017; World Health Organization, 2017). As antibiotics lose their effectiveness in the course of time due to the emergence of bacterial resistance, researches on antibiotics necessitate continuous improvements. However, the interest of pharmaceutical companies in antibiotic development dwindled over the last decades owing to scientific, regulatory, and economic difficulties (Brown and Wright, 2016; Luepke et al., 2016). Based on 10 different criteria including community and health-care burden, mortality, pipeline, prevalence of resistance, preventability in community and health-care setting, transmissibility, treatability and trend of resistance, 20 bacterial species among 25 antibiotic-resistant bacteria were classified into three priority groups as critical, high and medium priority. Multidrug-resistant *Mycobacterium tuberculosis* takes the global priority.

Over the quarter century after the first genome sequenced for pathogenic *Haemophilus influenzae* Rd in 1995, the advances in experimental and computational technologies have accelerated context-specific understanding of complex biological networks and the emergence of a new field of science, called systems biology (Fleischmann et al., 1995; Aggarwal and Lee, 2003). Metabolic reactions in conjunction with their corresponding metabolites and genes constitute a sophisticated cellular system inside the pathogens. This interconnection among genes, metabolites and reactions are converted into mathematical representation by using genome-scale metabolic modeling which is an ever increasingly used computational approach in the field of systems biology to elucidate pathogenic mechanisms along with their host interactions. Numerous pathogen-specific genome-scale metabolic models (GEMs) were reconstructed in the last two decades (Table 1). The key aspects such as predicting cellular and disease phenotypes, production of virulence factors, and evolution of antibiotic resistance related to human pathogens have been effectively studied by GEMs. In addition to identifying the cellular phenotype of a single pathogen, this modeling approach can be extended to interactions between pathogen and host cells (Jamshidi and Raghunathan, 2015).

**Table 1:** Reconstructed GEM examples of priority pathogens reported by World Health Organization (2017).

**Pathogen**	**Priority**	**Model name**	**Number of metabolites**	**Number of genes**	**Number of reactions**	**References**
*Mycobacterium tuberculosis*	Global	iNJ661	826	661	1,025	Jamshidi and Palsson, 2007
		GSMN-TB	645	726	856	Beste et al., 2007
		GSMN-TB 1.1	766	759	876	Lofthouse et al., 2013
		sMtb	929	915	1,192	Rienksma et al., 2014
		iOSDD890	961	890	1,152	Vashisht et al., 2014
		iSM810	723	810	938	Ma et al., 2015
		iEK1011	998	1011	1,128	Kavvas et al., 2018
*Acinetobacter baumannii*	Critical	AbyMBEL891	778	650	891	Kim et al., 2010
		iLP844	1,509	844	1,628	Presta et al., 2017
		iCN718	890	718	1,016	Norsigian et al., 2018
*Pseudomonas aeruginosa*	Critical	iMO1056	760	1,056	883	Oberhardt et al., 2008
		iMO1086	1,021	1,086	1,031	Oberhardt et al., 2011
		iPae1146	1,284	1146	1,493	Bartell et al., 2017
		iPau1129	1,286	1,129	1,495	Bartell et al., 2017
		iPAO1	3,022	1,458	4,265	Zhu et al., 2018
*Escherichia coli*	Critical	15 pathogenic strain	1,378–1,484	4,584–5,784	1,473–1,564	Vieira et al., 2011
*Klebsiella pneumoniae*	Critical	iYL1228	1,685	1,228	1,970	Liao et al., 2011
*Helicobacter pylori*	High	iCS291	403	291	388	Schilling et al., 2002
		iIT341	485	341	554	Thiele et al., 2005
*Salmonella typhimurium*	High	iRR1083	774	1,083	1,087	Raghunathan et al., 2009
		iMA945	1,036	945	1,964	AbuOun et al., 2009
		STM_v1.0	1,119	1,270	2,201	Thiele et al., 2011
		MetaSal	1,088	824	1„097	Hartman et al., 2014
*Staphylococcus aureus*	High	iSB619	571	619	641	Becker and Palsson, 2005
		iMH551	604	551	712	Heinemann et al., 2005
		iSA863	1,379	863	1,545	Mazharul Islam et al., 2020
*Campylobacter jejuni*	High	-	467	388	536	Metris et al., 2011
*Streptococcus pneumoniae*	Medium	iDS372	355	372	462	Dias et al., 2019
*Haemophilus influenzae*	Medium	iJE296	343	296	488	Edwards and Palsson, 1999
		iCS400	451	400	561	Schilling and Palsson, 2000

Essential genes are mandatory for the cellular growth, and their computational prediction plays a fundamental role in genome-scale modeling of pathogens. These genes are regarded as potential drug targets for killing pathogenic microorganisms. Instead of tedious and time-consuming experimental work, *a priori* computational analysis of gene essentiality and drug targeting by using constraint-based metabolic models can save considerable time and effort. The deletion of a single gene in the laboratory and observation of phenotypic change may take a few days or weeks; however, computational analysis of gene deletion is performed within seconds. An important point that need to be considered in the gene essentiality analysis for drug targeting studies, is essential genes be specific to pathogenic microorganisms.

Since their emergence two decades ago, GEMs have extended our knowledge toward system-level understanding of pathogenesis of microbial infections. They have provided irreplaceable contributions to elucidate antibiotic resistance, virulence, single pathogenic as well as pathogen-host interaction mechanisms. Pathogens change the key metabolic processes both in themselves and the host cell depending on the nutrient sources present in the infected host niche. Therefore, revealing cellular mechanisms of pathogenesis, identifying strain-specific biomarkers and developing new therapeutic approaches have great importance. Here, we reviewed the current knowledge of genome-scale metabolic modeling in pathogen cells with the specific examples mainly from WHO prioritization list as well as pathogen host interaction systems and its promising applications in the extension of curative strategies against pathogens for global preventative healthcare. GEMs integrated with genomic, proteomic and metabolomic data may be the first step toward the quantitative analysis of the pathogen metabolism, and thus can provide a remarkable benefit for the researchers as *a priori* guide in the drug target studies.

## Genome-Scale Metabolic Modeling and Analysis

**Figure 1:**
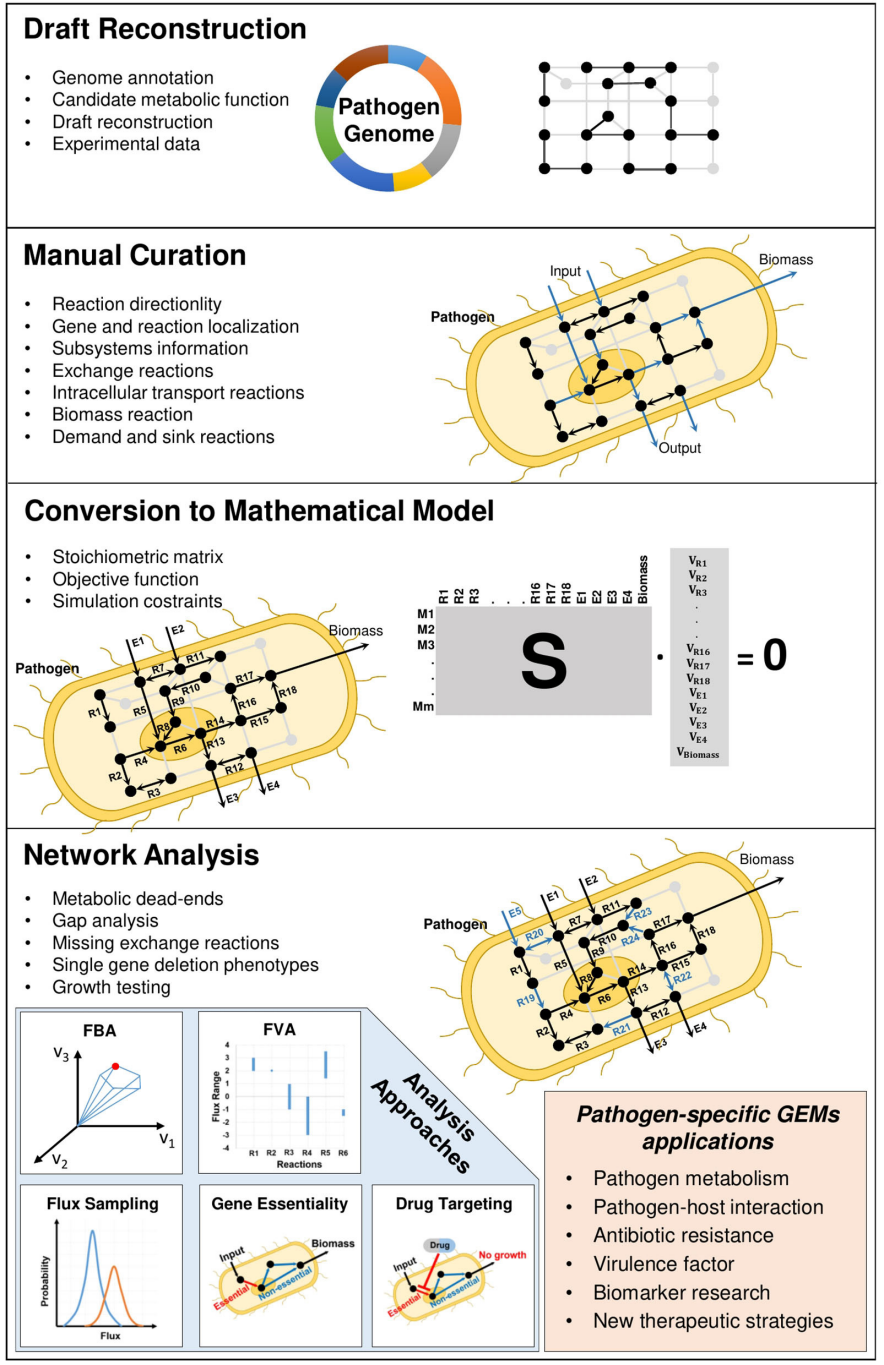
Reconstruction process of pathogen-specific GEMs.

First genome sequences were published for bacterial pathogens *Haemophilus influenzae* and *Mycoplasma genetalium* in 1995. Since then, continual improvements in genome sequencing technologies and their applications to genome analysis of pathogens have resulted in the comprehensive gene and cellular network information specific to microorganism of interest (Reed et al., 2006; Land et al., 2015). The cutting-edge technologies facilitated the understanding of complex cellular mechanisms behind the genomic variations in pathogens (Bryant et al., 2012). Starting with the genome annotation, the genome-scale metabolic modeling aims to reconstruct the mathematical representation of interconnected biochemical relationship among genes, reactions, and metabolites (Figure 1).

A crucial component of the pathogen-specific GEM reconstruction is the accurate inclusion of microbial metabolism to investigate cellular mechanism. In addition to common metabolic pathways, different pathogens have particular pathways, which are hallmark to expand models toward better understanding of pathogenic mechanisms. For example, mycolic acid is the unique component of the mycobacterial cell envelope and essential for the growth of devastating pathogen *M. tuberculosis* causing tuberculosis (Marrakchi et al., 2014). The metabolic reactions in mycolic acid biosynthetic pathway should thus be included in the GEM of *M. tuberculosis*. Moreover, virulence factors are the critical molecules in the infection mechanism and their synthesis pathways need to be incorporated into reconstructed GEM for valuable insight into virulence factor metabolism (Bartell et al., 2017).

In accordance with pathogen-specific data, validated experimentally or available in the literature, the localization of metabolic reactions into the different cellular compartments, the addition of transport reactions between the compartments inside the cell and the determination of biomass reaction with correct composition of its constituents (i.e., stoichiometric coefficients) play crucial roles in GEMs. When hundreds or thousands of metabolic reactions occurring inside the pathogenic cells and their literature-based distribution among different compartments are taken into consideration, the reconstruction process is extensively time-consuming and needs to be standardized. This reconstruction process was described by various studies (Francke et al., 2005; Rocha et al., 2008; Feist et al., 2009). A comprehensive protocol (Figure 1) with four main stages (draft reconstruction, manual curation, conversion to mathematical model and network analysis) in genome-scale metabolic modeling was published by Thiele and Palsson (2010). In a very recent publication, this protocol was extended toward developing multi-strain GEMs from a reference model (Norsigian et al., 2020).

Different tools and softwares have been developed to facilitate and accelerate metabolic reconstruction process (Hamilton and Reed, 2014; Mendoza et al., 2019). The biochemical databases such as KEGG, BRENDA, and MetaCyc have become remarkable sources in this reconstruction process to obtain collective information on genes, enzymes, reactions, metabolites and pathways (Kanehisa, 2000; Schomburg, 2002; Caspi et al., 2012). High-throughput phenotype microarrays technology (Biolog) provide clues toward the viability of the pathogenic microorganisms on hundreds of nutrient sources simultaneously (Bochner et al., 2001). Biolog phenotype microarrays take full advantage of respiration of the cells, detected by tetrazolium dye, as a reporter system. Therefore, in addition to the model refinement, Biolog data have been used in the validation of a wide range of GEMs (Oberhardt et al., 2008, 2011; Baumler et al., 2011; Liao et al., 2011; Metris et al., 2011; Bartell et al., 2017; Norsigian et al., 2018; Zhu et al., 2018).

Genome-scale metabolic models have many key advantages in the investigation of pathogenic processes. They allow to perform thousands of different infection scenario simulations in a very short time in an effective manner. Cellular phenotypes such as growth and virulence factor production are predicted by changing nutrient sources present in the host environment. Different infected locations within the human body show different nutrient distributions and thus different infection features and growth phenotypes that can be rapidly deduced from GEMs. They are highly cost-effective in gene deletion analysis. Thus, essential genes are examined by carrying out *in silico* simulations with gene deletion one by one in the genome-scale network. These predicted essential genes and their products are considered as putative drug targets to fight against pathogen of interest. Multi-omics data can be easily mapped to GEMs to investigate condition-specific pathogenicity. The integrated analysis of pathogen and host GEMs enables us to identify essential metabolic connections between host and pathogen and thus to unlock the mechanisms behind their interactions.

### Flux Balance Analysis

Flux balance analysis (FBA) is a frequently used constraint-based modeling approach to represent the possible behavior of microbial metabolism and plays an irreplaceable role in genome-scale metabolic modeling (Kauffman et al., 2003; Orth et al., 2010). FBA uses metabolic reaction stoichiometry together with the physiochemical and environmental constraints at steady state condition. Subsequent to conversion of genome-scale metabolic reconstruction to mathematical matrix format at steady state, FBA finds a flux distribution which maximizes the objective function. Stoichiometric coefficients of the metabolic reactions compose stoichiometric matrix (S), where the rows and columns are represented by metabolites and reactions, respectively. Metabolic fluxes constitute flux vector (*v*) of the metabolic reactions in the GEM. At steady state, a system of linear equations obtained from the metabolic network, is given by:

$$\begin{array}{l} \text{S}.\text{v}=0 \end{array}$$

Constraints, arising from reaction directionalities and experimental measurements, are imposed by arranging upper and lower limits. The exchange fluxes between the pathogen cell and environment are involved in accordance with the metabolites in the growth medium. Maximization of biomass is defined as objective function in order to simulate pathogen growth. The linear optimization problem used in FBA is summarized below:

$$\begin{array}{l} max\;f^{T}v\\ s.t\;S.v=0\\ v_{lower}\leq v\leq v_{upper} \end{array}$$

where f is objective function vector, the vector of coefficients assigning the cellular objective to each reaction.

### Flux Variability Analysis

Constraint-based modeling leaves open the possibility of alternate optimal solutions which mean that the same objective value can be achieved by a diverse set of flux distributions and the solution is not unique. Flux variability analysis (FVA) determines the possible range of flux quantities which is allowable with the given objective value (Mahadevan and Schilling, 2003). First, the value of the objective function that is maximization of growth in pathogenic microorganisms is computed by FBA. By adding and fixing calculated objective value in the model, a series of FBA/FVA is performed for each reaction in the GEM with the maximization and minimization objective function for allowable range of fluxes for each reaction. The reactions with the same minimum and maximum non-zero fluxes computed by FVA are essential in accomplishing certain objective. There are no alternative pathways for those in which these reactions exist and therefore these reactions and pathways are essentially involved in the GEM to succeed objective of interest. Mathematical formulation of FVA is given by:

$$\begin{array}{l} max\;v_{i}\text{ and }min\;v_{i}\\ \text{s}.\text{t S}.\text{v}=0\\ f^{T}v=Z_{obj}\\ v_{lower}\leq v_{i}\leq v_{upper}\text{ for }i=1,\ldots n \end{array}$$

where *Z*_*obj*_ is previously computed objective function value by using FBA.

Growth and metabolic state predictions are calculated by FBA and FVA. Flux variability analysis determines all possible alternate routes for growth of a microorganism and thus identifies a minimal set of reactions required for pathogen intracellular survival. FBA and FVA are used in testing and validation of the GEMs by comparing with the experimental data. The consistency between *in silico* growth simulations and experimental studies demonstrate the predictive power of the model.

### Flux Sampling

Flux sampling calculates all feasible solutions throughout the entire solution space in a statistical meaningful way (Price et al., 2004; Schellenberger and Palsson, 2009; Bordel et al., 2010; Herrmann et al., 2019). Sufficient and uniform data points are required for the accurate and unbiased analysis of the solution space. Both flux sampling and FVA computes feasible flux range; i.e., set of possible flux distributions. However, flux sampling gives additional information on probability of flux solutions. Different from FBA, flux sampling does not require an objective function. Therefore, it is an effective and alternative approach in analyzing the GEMs when certain objective of cell is not clear. Several algorithms were developed for flux sampling. Artificial centering hit-and-run (ACHR) algorithm calculates the random flux distributions by using the center estimate and random flux vector direction (Kaufman and Smith, 1998). Coordinate hit-and-run with rounding (CHRR) applies a rounding preprocessing to the anisotropic flux sets (Haraldsdóttir et al., 2017). *optGpSampler* provides an opportunity of use of large samples via parallel sampling to reduce computation process (Megchelenbrink et al., 2014).

### Gene Essentiality

Essential genes are necessary for completeness of metabolic network and proper functioning of the cell. They have crucial roles in the development of phenotypic features of microorganism. Their knockouts give rise to failure in the cellular metabolism and no growth condition eventually. Therefore, essential gene studies in pathogenic microorganisms gain further importance to fight against pathogens. Experimental analyses of essential genes are performed by different methods including random mutagenesis, targeted mutagenesis and knockdown approaches (Rancati et al., 2018; Gonyar et al., 2019). These studies constitute a significant platform for computational gene essentiality predictions by using pathogen-specific GEMs (Joyce and Palsson, 2007). Computationally predicted genes are compared with the experimentally obtained essential gene datasets for the predictive capacity of the GEMs. FBA is widely used in gene essentiality predictions. Genes are deleted from the model on an individual basis by setting the flux value of corresponding reactions to zero. The objective function, used in the optimization, is the maximization of biomass production. If the flux analysis results in no growth, the corresponding gene is predicted to be essential.

The formulation of the biomass reaction (equation) in the GEM holds a great importance (Thiele and Palsson, 2010). It should include accurate chemical compositions of the pathogen obtained from experimental studies. In addition to growth associated maintenance, required energy for macromolecular synthesis, the content of amino acid, nucleotide, lipid, soluble pool (polyamines, vitamins, and cofactors) and ions are required in the generation of biomass equation. The composition of the biomass reaction is critical for the analysis of essential genes. If a biomass precursor is not included in the biomass reaction, its synthesis reactions may not be necessary for the cellular growth and these reactions are regarded as non-essential with the associated genes in the computational analysis.

### Drug Targeting

Target identification is the primary and indispensable step in drug discovery and development to combat infection. Impairments in the metabolic functionalities of the investigated microorganisms give rise to cell death due to drug inhibition (Fischbach and Walsh, 2009). Conventional methods require an extensive and expensive experimental research to detect therapeutic targets. On the other hand, computational studies reveal a rapid and cost-effective alternative route by predicting novel targets, which are critical for cell growth. Over the last decade, genome-scale metabolic modeling has fulfilled an inevitable rise in the prediction of pathogenic drug targets. Within this scope, essential genes and their corresponding products, obtained from *in silico* analysis of pathogen-specific GEMs, are regarded as putative drug targets to inhibit cell survival.

The ideal drug target is the target with little or no harm to the host organism. Fungal sphingolipid pathways are different in many ways from their mammalian analogs in terms of enzymes and products. The glucosylceramide (GlcCer) lowering antifungal agents have an important potential to control and prevent infections. Acyhydrazones have been identified as the inhibitor of glucosylceramide synthase enzyme. When the glycosylceramide synthesis gene is deleted in *Cryptococcus neoformans*, the strain does not produce glycosylceramide and is avirulent in host organism. In this context, glucosylceramide (GlcCer) lowering agents or inhibitors of glycosylceramide synthesis can be considered as new opportunities to prevent fungal infections (Rittershaus et al., 2006; Raj et al., 2017).

### Other Methods

Constraint-based methods mentioned above are the most frequent techniques used in the analysis of pathogen-specific GEMs. Investigation of the *in silico* killing strategies of pathogenic bacteria is the main goal of the pathogen-specific GEMs; however, the maximum amount of production of valuable metabolites takes great importance in industrial microorganisms' GEMs with the minimum cost. Depending on the application of the GEMs, various approaches are also available in the literature. Among them, dynamic FBA (dFBA) was suggested for the study of the metabolic network dynamics since the classical FBA is used for steady state systems (Mahadevan et al., 2002). Minimization of metabolic adjustments (MOMA) and regulatory on-off minimization (ROOM) are used in the analysis of response to gene deletion or insertion (Segrè et al., 2002; Shlomi et al., 2005). Metabolic reactions controlled by the genes of interest are deleted from the GEM and the new reaction fluxes are optimized with the minimum metabolic change.

## Genome-Scale Metabolic Models of Pathogenic Microorganisms and Antibiotic Resistance

**Figure 2:**
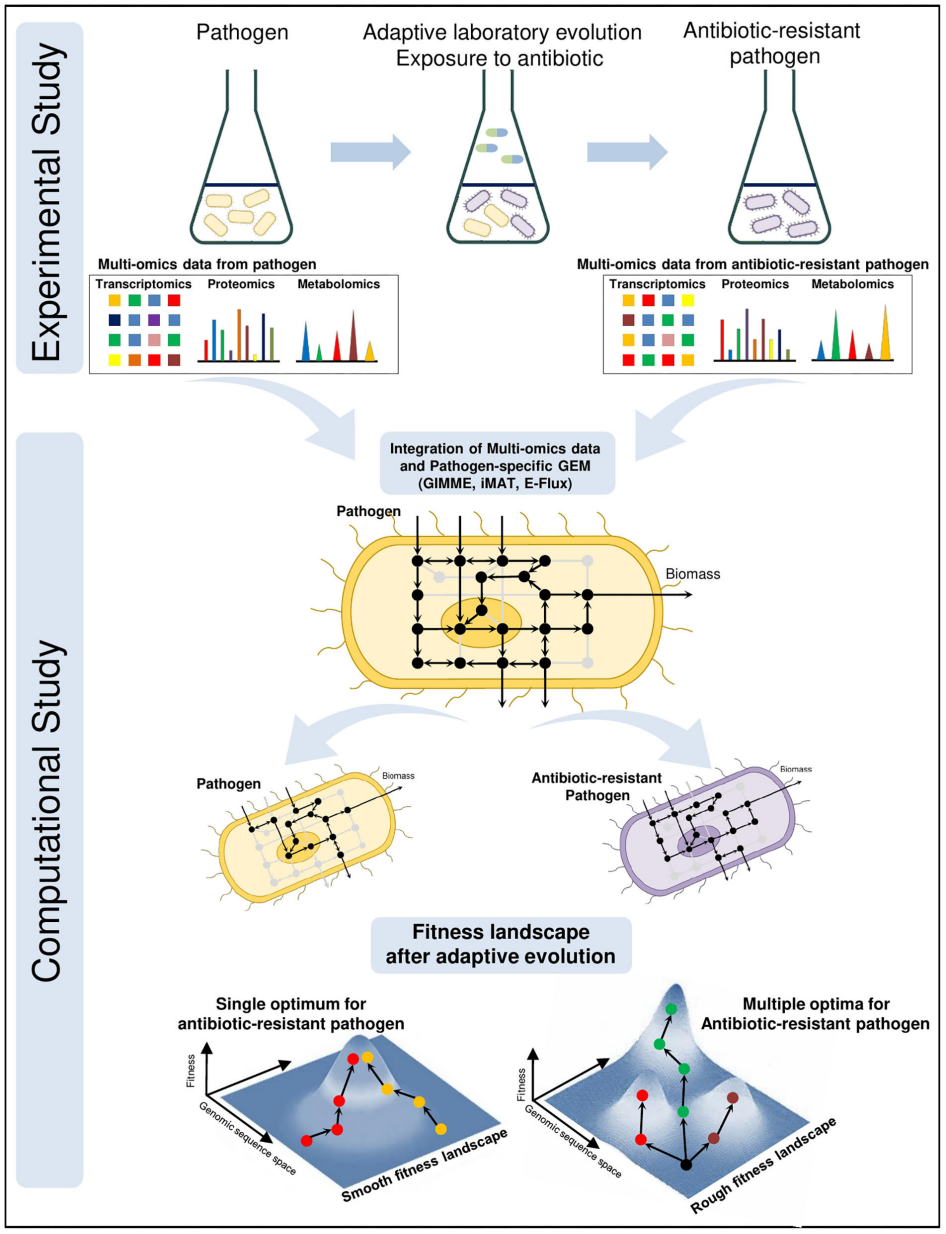
Integrated investigation of the antibiotic resistance by adaptive laboratory evolution (ALE) experiments and pathogen-specific GEMs. Multi-omics data are collected during the ALE experiments from wild-type and antibiotic-resistant pathogens at different time points. To elucidate the evolutionary response at system-level due to antibiotic pressure, high throughput omics data are computationally mapped onto the genome-scale metabolic network. Analysis of metabolic shift in the cellular metabolism and mechanism of antibiotic-resistant pathogenic GEMs facilitate the discovery of novel potential drug targets and treatment strategies against antibiotic-resistant pathogen. Fitness landscapes demonstrate the optimality in adaptive evolution of antibiotic resistance. Smooth fitness landscapes consist of a single optimum and regardless of the starting point evolutionary tendency converge to this optimum. There exist multiple optima on the rough fitness landscapes and evolutionary tendency diverge even from the same starting point.

Antibiotic resistance is a growing problem threatening global health. Development of promising novel treatments require a complete understanding of resistance mechanisms. For this purpose, adaptive laboratory evolution experiments are frequently used where the pathogens are treated with certain antibiotics and tested whether to develop resistance (Conrad et al., 2011; Dragosits and Mattanovich, 2013; Zampieri et al., 2017; Dunphy et al., 2019). Multi-omics technologies, including transcriptomics, proteomics and metabolomics, have become crucial components of these experiments since they provide simultaneous measurements of thousands of gene expressions, proteins and metabolites, respectively. Muti-omics data are collected from the wild-type and antibiotic-resistant pathogens at various time points. Then, they are integrated with the pathogen-specific GEMs for system-level understanding of pathogenic shifts due to antibiotic resistance (Figure 2). The integration process, that maps high throughput omics data onto high-connected metabolic network, facilitates the prediction of feasible flux distributions throughout pathogenic metabolism. Consequently, the novel therapeutic strategies are proposed to combat antibiotic resistance as well as infection.

Several applications of GEMs in antibiotic resistance have been published by the researchers around the world (Dunphy and Papin, 2018). Recently, the flux distributions and metabolic changes of streptomycin resistant and chloramphenicol resistant *Chromobacterium violaceum* were studied by using its specific GEM (iDB858) and metabolomics data (Banerjee and Raghunathan, 2019). FVA was carried out to predict the metabolic reprogramming due antibiotic pressures. Chloramphenicol resistance enhanced acetate production, whereas streptomycin resistance resulted in increased secretion of both acetate and formate. NAD/NADH ratio of streptomycin resistant population growing on glucose was calculated to be higher than that of chloramphenicol resistant population (2.47–0.28). Further works on this issue will be discussed under the related pathogenic bacteria below.

### Mycobacterium tuberculosis

*M. tuberculosis* is one of the most studied pathogens in genome-scale metabolic modeling due to its global priority. The first GEMs published for this pathogen are iNJ661 and GSMN-TB (Beste et al., 2007; Jamshidi and Palsson, 2007). Genome annotation of *M. tuberculosis* H37Rv was used as the starting point in iNJ661 model reconstruction (Cole et al., 1998). However, GSMN-TB model was initiated with previously developed *Streptomyces coelicolor* genome-scale metabolic model (Borodina et al., 2005). *M. tuberculosis* specific reactions obtained from literature and biochemical databases were added to both models throughout the reconstruction processes. Instead of starting with annotated genome, previously developed genome-scale metabolic models can also be used as a draft in the reconstruction process. Therefore, these initial models became a starting point for the most of the subsequent *M. tuberculosis* genome-scale metabolic models. More than 10 genome-scale models were developed for this pathogen with divergent representations around the world by extending previous ones (Lofthouse et al., 2013; Rienksma et al., 2014, 2018; Vashisht et al., 2014; Ma et al., 2015). They were standardized and updated with a new model iEK1011, including 1228 reactions, 1,011 genes and 998 metabolites (Kavvas et al., 2018). Recently, eight *M. tuberculosis* GEMs were systematically evaluated for accurate model selection (López-Agudelo et al., 2020). By taking genetic information, network topology, blocked reactions, mass and charge balance reactions and gene essentiality predictions into consideration, iEK1011 and sMtb2018 were described as the best performing *M. tuberculosis* GEMs. These two GEMs were further improved and named iEK1011_2.0 and sMtb2.0, respectively. These updated versions allow comprehensive *in silico* investigation of the effects of various environmental and genetic conditions on *M. tuberculosis* metabolism.

Jamshidi and Palsson (2007) analyzed essentiality of glycerol and non-essentiality of glucose in *M. tuberculosis* model iNJ661 by using FVA at maximum biomass production. Glycerol transporter and glycerol kinase in glycerol metabolism were computed as non-zero flux values with no flexibility. This shows that glycerol-3-phosphate production via glycerol kinase is required for membrane and fatty acid metabolism. Beste et al. (2007) investigated *in silico* slow growth mechanism of *M. tuberculosis* by using GSMN-TB. The reactions in the glyoxylate shunt were predicted to be significantly changed due to the slow growth of organism. A high increase was computed in the isocitrate lyase reaction flux. This observation proposed a hypothesis of a key role of isocitrate lyase to sustain growth at the slow growth condition. The activity of isoctrate lyase was measured at slow and fast growth rates experimentally. Consistent with the simulations, the activity of this enzyme was twofold higher in the slow growing cells.

The mechanism of antibiotic resistance evolution in *M. tuberculosis* was investigated by means of *in silico* iEK1011 (Kavvas et al., 2018). Antibiotic-specific pressures were imposed by using an associated metabolic objective function. For example, decaprenylphosphoryl-β-D-arabinose (DPA) production was elevated by *ubiA* mutations, which cause ethambutol resistance. Consequently, the maximization of DPA production was used as the objective function to simulate antibiotic resistance evolution due to ethambutol (Safi et al., 2013). In order to incorporate this evolution into *in silico* iEK1011 model, the minimization of mycothiol production was selected for ethionamide and the maximization of tetrahydrofolate and L-alanine production were chosen as objective functions for para-aminosalicylic acid and d-cycloserine, respectively (Vilchèze et al., 2008; Zheng et al., 2013; Desjardins et al., 2016). The computational analyses of *in vivo* and *in vitro* conditions were performed by FVA, and an important effect of L-alanine due to correlation between the ethambutol and d-cycloserine fluxes was unraveled. These two antibiotics may be less effective *in vivo* owing to the presence of L-alanine.

### Acinetobacter baumannii

An important high threat pathogens in hospitals is *A. baumannii*, causing various infections covering pneumonia, blood-stream, urinary-tract and wound infections (Dijkshoorn et al., 2007). First GEM for multi-drug resistant *A. baumannii* AYE is AbyMBEL891, reconstructed from genome annotation data integrated with literature and biological databases (Vallenet et al., 2008; Kim et al., 2010). Similarly, genome-scale metabolic model iLP844 was developed for *A. baumannii* ATCC 19606, which was initiated with a draft model from genome annotation by using Kbase and then manually curated (Davenport et al., 2014; Presta et al., 2017; Arkin et al., 2018). The publications of new studies lead to the inclusion of new knowledge into the model and also to the validation of the model with more experimental work enhancing its prediction quality. In accordance with this purpose, AbyMBEL891 model was updated with the new data produced in experiments and standardized into a new model, iCN718, to provide more accurate representation of the microorganism (Norsigian et al., 2018). iCN718 was used in the simulation of growth behavior and the development of strain-specific GEMs of 74 other *A. baumannii* strains.

The role of reaction reversibility on gene essentiality was simulated using *in silico* AbyMBEL891 model of *A. baumannii* (Kim et al., 2010). Ornithine carbamoyltransferase and argininosuccinate synthase reactions are experimentally demonstrated to be essential in arginine biosynthesis and cell growth in the *A. baumannii* (Dorsey et al., 2002). These reactions were also computationally estimated to be essential on condition that a series of alternative path reactions toward arginine production were set irreversible. If the reactions in alternative route were set as reversible, two essential reactions became non-essential and AbyMBEL891 lost the capability to synthesize arginine. Thus, *in silico* AbyMBEL891 model gives consistent simulation results with *A. baumannii* phenotype and provides a valuable use in reaction reversibility.

Similar to essential genes, the metabolites whose absence leads to no growth are considered essential metabolites (Kim et al., 2010). They are predicted by using flux balance analysis and removing each metabolite out of the cell to explore their effect on cell growth. Computational investigation of an essential metabolite in the GEM is performed by the deletion (zero flux values) of all outgoing reactions and allowing non-zero flux values of the incoming reactions associated with the interested metabolite (Kim et al., 2007). Kim et al. (2010) presented essential metabolite filtering method (EMFilter) to discover effective drug targets for *in silico* AbyMBEL891. EMFilter includes four steps that are removal of currency metabolites, selection of essential metabolites existing in more than three reactions, removal of metabolites present in human metabolic network and removal of metabolites related with genes which have human homologs. In order to avoid drug interference with any of the human enzymes and identify multi-drug target, EMFilter narrowed 211 essential metabolites down to 9 metabolites for drug targeting by using these four steps. The enzymes catalyzing the outgoing reactions associated with essential metabolites were predicted to be final candidate drug target. D-glutamate and 4-aminobenzoate were predicted as essential metabolites *in silico* and they have critical roles in the biosynthesis of bacterial cell wall and folate, respectively (Lundqvist et al., 2007; Valderas et al., 2008). Six essential metabolites were also predicted to be essential for four pathogens among *A. baumannii, E. coli, H. pylori, M. tuberculosis, P. aeruginosa*, and *S. aureus*. Therefore, the enzymes catalyzing the reactions in which these six essential metabolites are involved could be regarded as broad-spectrum drug targets due to the fact that a broad-spectrum antibiotic acts against a wide range of pathogenic bacteria.

The colistin-resistant *A. baumannii* metabolism was studied by GEM to determine new putative drug targets (Presta et al., 2017). The *in silico* model iLP844 was integrated with transcriptomic response to colistin, sampled at 15 and 60 min after its exposure (Henry et al., 2014). The integration was performed by using MADE (Metabolic Adjustment by Differential Expression) method which maps gene expression data onto genome-scale metabolic network (Jensen and Papin, 2011). Essential genes were computed at 15 and 60 min with and without colistin exposure to analyze the shifts in gene essentiality. In addition to 66 essential genes shared between with and without colistin exposure, 21 and 17 condition-specific essential genes were predicted for 15 and 60 min data, respectively. Following colistin exposure, some essential genes became non-essential and vice versa. These results demonstrated changes in gene essentiality patterns due to colistin and consequently significant drug targets for *A. baumannii* ATCC 19606 strain. Potential drug candidates should be pathogen-specific and should not have orthologs in human to avoid side-effects. Subsequent to BLAST search, they defined four condition-specific and 46 general essential genes without human orthologous (Altschul et al., 1990). To study colistin resistance mechanism, loss of lipopolysaccharide (LPS) production approach was implemented with the transcriptomic data sampled at 60 minutes (Moffatt et al., 2010). LPS component was removed from biomass reaction in iLP844 to simulate LPS deficit *A. baumannii*. They observed 54 shared essential genes in the presence and absence of colistin resistance at 60 min. Eighteen genes switched from non-essential to essential. By applying BLAST search, they demonstrated that five essential genes do not have orthologs in human and these can be considered as specific targets in combination therapy with colistin in LPS deficient strain of *A. baumannii*.

### Pseudomonas aeruginosa

Pathogenic genome-scale metabolic modeling can potentially be used for comparative analysis and drug assessment. Genome-scale metabolic model iMO1056 was developed for *P. aeruginosa* PAO1 from annotated genome, published studies and databases (Stover et al., 2000; Oberhardt et al., 2008). Three years later, iMO1056 was updated with iMO1086 and reconciled with non-pathogenic *P. putida* model (iJP962) by the same research group for comparative analysis of these two strains for understanding the pathogenicity (Oberhardt et al., 2011). To reflect different mechanisms in these two microorganisms, a number of unique reactions and pathways were identified including naphthalene and anthracene degradation, phenylalanine metabolism and benzoate degradation in *P. putida* and pyrimidine, purine and beta-alanine metabolism in *P. aeruginosa*. The *in silico* iMO1056 was used in the study of multiple targets in *P. aeruginosa* and 41 putative targets were suggested (Perumal et al., 2011). Recently, Bartell et al. (2017) presented two GEMs for *P. aeruginosa* strain PAO1 and *P. aeruginosa* strain PA14 (iPae1146 and iPau1129, respectively) to provide insight into the relationship between virulence factor production and growth. These two *in silico* models included 112 and 108 virulence-associated genes, respectively. The genes only critical for virulence factor production, growth and both were analyzed and thus non-obvious links between virulence factor production and growth were observed. iMO1056 and Opt208964, an automatically generated GEM by Model SEED, were used as initial drafts in the development of iPAO1, which is then extensively curated using the literature and databases toward the study of polymyxin treatment in *P. aeruginosa* metabolism at the systems level (Henry et al., 2010; Zhu et al., 2018). Together with other biochemical databases, *Pseudomonas aeruginosa* specific database PseudoCAP provides valuable metabolic information for GEM reconstruction of this organism (Winsor et al., 2005).

Oberhardt et al. (2011) performed comparative pathway flexibility analysis of pathogenic *P. aeruginosa* and non-pathogenic *P. putida* by using *in silico* iMO1086 and iJP962, respectively. Three sulfur-associated pathways, which are sulfur metabolism, taurine and hypotaurine metabolism, and cysteine and methionine metabolism, showed more flexibility in *P. aeruginosa* than in *P. putida*. This finding demonstrated significant probability of sulfur-associated pathways behind the distinct phenotypes. Enhanced flexibility of *P. aeruginosa* was also simulated in terms of nitrogen metabolism and the demand reactions of virulence factor. *P. aeruginosa* is able to carry out denitrification in microaerobic condition which can be seen in lung infections of cystic fibrosis patients (Eschbach et al., 2004), whereas *P. putida* cannot carry out denitrification. Increased flexibility simulation of *P. aeruginosa*, in comparison with *P. putida*, is in agreement with the known role of denitrification in virulence.

The essential genes in *Pseudomonas aeruginosa* were investigated for both virulence factor synthesis and growth by using *in silico* mPA14 and comparing 46 shared essential genes for biomass production and synthesis of at least one virulence factor (Bartell et al., 2017). Seven genes (*aacA, aacB, aacC, aacD, fabB, fabD*, and *fabG*) in fatty acid and phospholipid metabolism were predicted to be essential for growth and production of at least eight virulence factors. Growth-essential genes in aromatic amino acid synthesis (*aroB, aroC, aroE*, and *aroK*) were predicted to be essential for the synthesis of six virulence factors which are chorismate, pyocyanin, 1-carboxyphenazine, salicylate, dihydroaeruginoic acid and pyochelin. Moreover, the interconnectivity for each knockout was computationally researched by plotting virulence factor vs. growth inhibition compared to wild-type simulations. Uncertain connections between growth and virulence factor production were determined from the distribution of data points on plots. Several growth-essential genes partially hindered virulence factor synthesis. The virulence factor-related essential genes were significantly altered and not correlated with pathway complexity.

Investigation of metabolic response of pathogenic *P. aeruginosa* to polymyxin B treatment unlocked its effect on bacterial metabolism with the help of genome-scale metabolic model iPAO1 (Zhu et al., 2018). RNA-seq data obtained in the presence and absence of polymyxin B were combined with the *in silico* model by using E-Flux method constraining fluxes as a function of the gene expression level (Colijn et al., 2009). Several pathways involved in central, amino acid and fatty acid metabolisms were found to be significantly perturbed because of polymyxin B treatment which enhanced oxygen uptake and decreased the growth rate. In TCA cycle, NADH production and the fluxes from citrate to fumarate were increased. The reduction in biomass production due to polymyxin treatment resulted in the downregulation of fluxes in LPS, GPL and peptidoglycan biosynthesis. Spermidine biosynthesis enhanced with the increased expression level of *sdeD* and *speE* encoding S-adenosyl-L-methionine decarboxylase and spermidine synthase, respectively.

### Escherichia coli

Due to its genetic simplicity as well as its history in a variety of infections, *Escherichia coli* is one of the most extensively studied microorganisms in genome-scale metabolic modeling. Based on the genome sequence, the first GEM (iJO660) for *E. coli* was reconstructed for K-12 MG1655 commensal and common laboratory strain (Blattner et al., 1997; Edwards and Palsson, 2000). Over the last two decades, several updates were published to expand the understanding of bacterial mechanism via *in silico* network of *E. coli* (Reed et al., 2003; Feist et al., 2007; Orth et al., 2011; Monk et al., 2017). Besides the commensal strains, there also exist pathogenic strains of *E. coli* which brings about different intestinal and extraintestinal infections (Kaper et al., 2004). So as to reveal further insight into evolution mechanism *in silico*, Baumler et al. (2011) developed six strain-specific genome-scale model of *E. coli* (two enterohemorrhagic (EHEC), two uropathogenic (UPEC) and two commensal strains) by means of pangenome and core GEM. In addition to several pathogen specific reaction deletions, eight new reactions unique to EHEC strains were added, which are fructose synthetase, gentisate 1,2,-dioxygenase, perosamine synthetase, salicylate hydroxylase, sucrose transport, urease, tellurite reduction and UDP-N-acetylglucosamine 4-epimerase reactions. UPEC strains included only one addition of propionate CoA-transferase reaction in common. Furthermore, one unique reaction was added to each UPEC strain; hydroxy-pyruvate reaction for UTI89 strain and galactose isomerase reaction for CFT073 strain. The comperative simulations of strain-specific *E. coli* models resulted in variations of biomass yields on glucose due to the strain-specific metabolic reactions.

By increasing the number of the strains investigated, the diversities of the commensal and pathogenic *E. coli* strains were broadened (Vieira et al., 2011; Monk et al., 2013). Vieira et al. (2011) developed a genome-scale metabolic network for the analysis of core and panmetabolism in 29 *E. coli* strains including 21 pathogenic strains and eight commensal strains, which cover six *Shigella* strains. The reconstructed strain-specific GEMs resulted in 1,545 and 885 reactions in panmetabolism and core metabolism, respectively. The removal of *Shigella* strains did not significantly change the reactions in panmetabolism (1,545–1,543); however, increased number of reactions in the core metabolism to 1,065 from 885. This increase in the number of the reactions in the core metabolism among *E. coli* strains indicates a conserved metabolism in *E. coli*. The reactions absent from *Shigella* core metabolism were distributed in various pathways including D-allose degradation, phenylethylamine and phenylacetate degradation, and biosynthesis pathways related amino acids, nucleotides and fatty acids. Another strain-specific GEM was built for 55 strains of *E. coli* and *Shigella* to shine light on adaptations to diverse environments (Monk et al., 2013). This study increased the reactions in core and panmetabolism to 1,773 and 2,501, respectively. Similarly, strain-specific GEMs of 64 strains of *S. aureus* were reconstructed for elucidating the metabolic capabilities associated with the pathogenity (Bosi et al., 2016).

### Klebsiella pneumoniae

In the reconstruction and analysis of iYL1228 for *K. pneumoniae* MGH 78578, FBA was performed for the growth phenotype predictions on different nutrition media including carbon, nitrogen, phosphorus and sulfur to compare with the Biolog data (Liao et al., 2011). The discrepancies between computational results and Biolog data were taken as advantages toward the curation of the model by pioneering and adding experimentally supported metabolic reactions. Subsequent to refinement, iYL1228 was able to predict 84% of phenotypes among 171 growth conditions. In the same study, in addition to *in silico* viability predictions, experimental growth rates of nine carbon sources under aerobic conditions were investigated and two of them (citrate and myo-inositol) were predicted significantly higher than those of experimental results. After adaptive evolution of *K. pneumoniae* to myo-inositol by serial passage, the experimental growth rate increased and the rate of growth error decreased from 80% to 24%. Hence, GEMs can also give remarkable clues toward uncovering adaptive evolution mechanism. In another study, the *in silico* iYL1228 was used as a platform to reconstruct GEMs for 22 *K. pneumoniae* strains (Norsigian et al., 2019). These models were manipulated for the investigation of growth capabilities on carbon, nitrogen, sulfur and phosphorus sources. Carbon, nitrogen and sulfur simulations varied across the strains; however, phosphorus results were largely remained the same.

The screening of putative drug targets in *K. pneumoniae* was studied by iYL1228 with the improved biomass reaction (Cesur et al., 2020). So as to mimic the host microenvironment, the growth simulations were performed by using FBA in three different conditions including human body fluid, sputum-macrophage, and generic host media. Gene-centric (essential genes) and metabolite-centric (essential metabolites) approaches were employed to identify whether they are indispensable for bacterial metabolism or not. Since potential drug targets are required to exist in only pathogen metabolism, homology analysis of essential genes was performed and presence of essential metabolites among human metabolites were examined to eliminate possible side effects in the host metabolism. Further *in silico* prioritization approaches such as subcellular localization, druggability, antibiotic resistance, virulence and broad-spectrum analysis were performed to come up with more effective targets. 2-dehydro-3-deoxyphosphooctonate aldolase (KdsA) was identified as the highest-ranked putative drug target satisfying virulence, druggability and broad-spectrum criteria and ZINC95543764 was suggested as a potential *Klebsiella* inhibitor in gene-centric approach. The enzymes related to essential metabolites were suggested as putative drug targets in metabolite-centric approach. These are enoyl-(acyl carrier protein) reductase (FabI) in fatty acid metabolism, riboflavin synthase subunit α (RibC) and riboflavin synthase subunit β (RibH) in riboflavin metabolism, and penicillin-binding protein 1A-C (PBP 1A, PBP 1B, and PBP 1C) in the peptidoglycan biosynthesis. PBP 1A-C were found to be synthetic lethal and the remaining three were essential.

### Salmonella typhimurium

Salmonellae are gram-negative bacterial pathogens with a wide host range, causing millions of human infections and hundreds of thousands of deaths per year worldwide. The serovars of *Salmonella* differ in their antimicrobial host specificity, resistance profiles, and virulence phenotypes. For example, *S. typhimurium* is the leading cause of human gastroenteritis and have more than 2000 serovars. An *in silico* strain-specific metabolic reconstruction was performed for 410 *Salmonella* strains and the metabolic capabilities on minimal media with more than 500 different growth-supporting nutrition sources including carbon, nitrogen, phosphorous, and sulfur were compared across the *Salmonella* genus in aerobic and anaerobic conditions (Seif et al., 2018). One thousand nine hundred thirteen metabolic reactions associated with 1,013 genes and 1,407 metabolites are shared across all 410 strains. The common strains mostly differ in their capability to utilize D-tagatose, myo-inositol, 2,3-diaminopropionate, allantoin, D-galactonate, and 2-aminoethylphosphonate. All 6 Typhi strains were predicted to lack the capability to utilize L-idonate (an available nutrient source in the gut) due to the absence of *idnD* and *idnO* and show lack of fitness (inability of the organism to thrive in a competitive environment). A total of 21 catabolic pathways like the utilization of D-tagatose, L-xylulose, D-xylose, deoxy-D-ribose, L-idonate, D-glyceraldehyde, and allantoin, that form part of the host's diet and/or exist in the intestinal environment, contributed to *Salmonella* fitness during intestinal infection. The compositional differences in the intestinal vs. extraintestinal milieu of the host may reflect differences in pathogenicity of different *Salmonella* types.

The *in silico* model of *S. typhimurium* (iRR1083) provided a suitable platform to analyze the role of reactions important in infection and pathogenesis, such as those required for drug efflux, proton pumps, inhibitory effects of antibiotics, mechanisms for reactive nitrogen species (RNS) and reactive oxygen species (ROS) resistance (Raghunathan et al., 2009). Two hundred (out of 1,083) metabolic genes were predicted as essential. Gene essentiality analysis was extended to virulence predictions study in such a way that *Salmonella* mutants defective in essential and non-essential genes were considered as avirulent (no growth) and virulent (can grow) in the host cells, respectively. Of the 24 *in vivo* essential genes from the literature, the *in silico* analysis correctly predicted 22 virulence characterizations. For example, AceA, which was predicted as non-essential and mutants defective in *AceA* are virulent. AceA is required only for chronic and not for acute *Salmonella* infections (Fang et al., 2005). The double deletion of two genes (*ackA* and *pta*) responsible in acetyl phosphate formation lead to virulent strain. The fatty acid degradation genes *fadA, fadD/fadE* genes have been implicated for virulence in chronic *Salmonella* infection, however the model predicted the presence of glucose or pyruvate but not acetate or short chain fatty acids during the early stages of infection (Fang et al., 2005) and these fad genes are not being operational during the early stages of infection. Furthermore, simultaneous upregulation of *cyoE* and *cyoA* (succinate dehydrogenase and electron transport chain genes) was reported suggestive of an aerobic condition *in vivo*, during the early stages of infection (Karatzas et al., 2008). Increased levels of F_1_F_0_ ATP synthase subunits, heme biosynthesis, upregulated histidine biosynthesis (hisC, hisD, hisG) predictions were consistent with the literature (Eriksson et al., 2003; Shi et al., 2006; Karatzas et al., 2008). The *in silico* model accurately predicted that catalase (*katE* and *katG*) mutants and super oxide dismutase mutants (*sodA* and *sodCII*) are virulent, confirming the pathogens have multiple defenses to oxidative stresses. On the other hand, the model predicted that mutants defective in aromatic amino acid biosynthesis are avirulent.

### Staphylococcus aureus

Taking the evolution due to drug resistance into consideration, GEMs for 13 multidrug-resistant *S. aureus* strains were reconstructed by using genome annotation, functional pathway analysis and comparative genomics approaches (Lee et al., 2009). The number of metabolic reactions in these GEMs varied between 1,444 and 1,497, and over 90% of these metabolic reactions, metabolites and enzymes were shared in common by all 13 strain-specific GEMs. Several amino acid biosynthesis pathways were involved in all strains, but L-histidine, L-serine, L-homocysteine and L-asparagine pathways were absent in all 13 strains. Fatty acid biosynthesis genes (*fabG, fabA, fabZ, fabI, fabK, fabH*, and *fabF*) were present in all strains. All these genes, exluding *fabI*, were determined as essential. The analysis of single unconditionally essential enzymes (essential in rich medium and computed without any limitation on fluxes of uptake reactions) resulted in 70 essential enzymes in one or more of 13 strains and 44 in all *S. aureus* strains. Of the 44 shared essential enzymes, minimum six of them were experimentally supported in *S. aureus* including transketolase, hydroxyl-methylbilane synthase, methionine adenosyltransferase, UDP-N-acetyl-glucosamine 1-carboxyvinyltransferase, protein N (pi)-phospho-histidine-sugar phosphortransferase and acetyl-CoA carboxylase (Forsyth et al., 2002). Simultaneous inactivation of two enzymes in the GEMs could lead to lethality or no growth while their single (one-at-a-time) knockout does not result in lethality. Of the 54 synthetic-lethal pairs of enzymes, 10 pairs were predicted in all 13 strains. Among them, prephenate dehydrogenase and arogenate dehydrogenase pair is involved in amino acid biosynthesis, and UDP-N-acetylglucosamine pyrophosphorylase and phosphoglucosamine mutase pair in cell wall metabolism. The synthetic lethal sets, i.e., the combinations of genes, which when simultaneously deleted, abolish growth *in silico*, allow to decipher complex interactions in reconstructed metabolic networks (Thiele et al., 2011).

Strain-specific GEMs enabled phenotype predictions via metabolic flux distributions of 64 *S. aureus* strains in more than 300 growth conditions (Bosi et al., 2016). The majority of the reactions different between the core and panmodels were identified in amino acid biosynthesis, and these diversities may lead to adaptation of different strains to different nutritional conditions. For all 64 *in silico* strain-specific models, vitamins B1 (thiamin) and B3 (niacin) were additionally required to grow in glucose minimal media. The thiamin and niacin auxotrophy are due to absence of the pathways converting tyrosine to thiamin and nicotinate-nucleotide diphosphorylase, respectively. The prediction of growth capabilities on alternative sources (carbon, nitrogen, phosphorous, and sulfur) indicated 238 nutrients including glucose and glycerol as carbon sources and arginine as nitrogen source are used by all 64 *in silico S. aureus* strains. Among other strain-specific nutrients, both uracil and thymidine were predicted as nitrogen source for 42 strains, and dulcose and inosine as carbon source for 42 and 13 strains, respectively. The simulations of growth resulted in the presence of two virulence factors (staphylokinase and IgG binding protein A precursor) and the ability to catabolize maltotriose distinguished human-associated strains from livestock-associated strains. Thus, the simulations based on genome-scale metabolic modeling allow the classification of *S. aureus* strains according to the presence of virulence factors.

### Biothreat Agents

The Centers for Disease Control and Prevention (CDC) evaluated the potential threats from various microorganisms and classify them into three categories (Rotz et al., 2002). Category A consists of the pathogens that are considered the highest risk with mass casualties for public health and necessitates broad-based preparedness efforts, Category B have some risk for large-scale dissemination, and Category C pathogens are emerging infectious disease threats. The discovery of antibiotics against these biological agents may not be economically feasible; however, it is clear that preventative and curative healthcare solutions are urgently needed. Toward this end, in addition to drug repurposing strategy, where the existing medications are searched for new successful treatments, utilization of antimicrobial peptides (AMPs) potentially constitute therapeutic options for high threat pathogens (Findlay et al., 2016; Farha and Brown, 2019; Miró-Canturri et al., 2019). The combinatorial therapeutic potential of one 24-amino acid AMP (WLBU2) and three early generation antibiotics (tigecycline, minocycline and novobiocin) were studied for two Category B pathogens (*Burkholderia mallei* and *Burkholderia pseudomallei*) and three category A pathogens which are *Yersinia pestis Bacillus anthracis* and *Francisella tularensis* causing plague, anthrax, and tularemia, respectively (Cote et al., 2020). By using the checkerboard MIC titration assays, it was observed that the combination of novobiocin-AMP enhanced the sensitivity of all five biological agents of interest. The tetracycline-peptide combinations increased the sensitivities of four agents including *Y. pestis, F. tularensis, B. anthracis, and B. pseudomallei*. The results showed that combinations of antibiotic-AMP are useful tools to fight against biological threat agents. Antimicrobial action mechanisms of AMPs can occur both directly and indirectly (Mahlapuu et al., 2016; Deslouches and Di, 2017). The direct cytotoxicity of AMPs electrostatically affects the bacterial outer surface by disrupting a phospholipid membrane. Subsequent to entry of AMPs into the cell, they interfere with critical intracellular processes including RNA and protein synthesis. Indirect AMP actions occur through immunomodulatory activities such as chemotactic stimulation, immune cells differentiation, inflammatory cell response regulation and cell death pathways.

A GEM study (iPC815) on *Yersinia pestis* revealed essential genes that might contribute to propose possible targets for antibiotic development (Charusanti et al., 2011). The largest group of predicted essential genes was found within amino acid transport and metabolism followed by nucleotide and coenzyme metabolism, cell membrane biogenesis and lipid metabolism. Several essential genes in these metabolisms were also identified as essential in *in silico E. coli* and *S. typhimurium* GEMs (Feist et al., 2007; Thiele et al., 2011). The predicted essential genes reflect a likely overlap among these three pathogens (*Y. pestis, E. coli* and *S. typhimurium*), the family of Enterobacteriaceae. The shared essential enzymes should be further exploited for the development of broad-spectrum antibiotics that can be used against this group of human pathogens.

The *in silico* analysis of *Francisella* strain demonstrated that *Francisella tularensis* undergoes significant changes of its metabolism upon its entry into the host cell (during intracellular growth) (Raghunathan et al., 2010). A switch from oxidative metabolism (TCA cycle) in the initial stages of infection to glycolysis, fatty acid oxidation, and gluconeogenesis during the later stages was found by flux balance and variability analyses. Moreover, the accumulation of 5-aminoimidazole-4-carboxamide ribonucleotide (AICAR) was found as a regulator of fructose bis phosphatase (*fbp*) gene in *Francisella* and the adenylyosuccinate lyase gene that catalyzes its formation, is a condition independent lethal gene, essential for survival and are proposed as a potential drug target. Thus, the predicted accumulation of AICAR in the host (macrophage) revealed a role as a potential master regulator during infection.

A multidisciplinary approach was applied to *F. tularensis* to select a group of enzymes as drug targets and ~20,000 small-molecule compounds were screened to list potential inhibitors against these targets (Chaudhury et al., 2013). A set of 40 candidate compounds was identified for antimicrobial activity against *F. tularensis*. Moreover, based on FBA predicted inhibition of NAD^+^ synthase (NadE), pantetheine-phosphate adenylyltransferase (CoaD), chorismate synthase (AroC) and sedoheptulose 7-phosphate isomerase (LpcA) enzymes, that are important in various metabolic pathways, these enzymes were suggested as potential drug targets. NadE takes part in NAD^+^ biosynthesis, CoaD in coenzyme A biosynthesis, AroC in phenylalanine, tyrosine and tryptophan biosynthesis and LpcA in lipopolysaccharide biosynthesis. These four enzymes are putative drug targets and the experimental validation of their enzymatic inhibition is an important step in the development of candidate antimicrobial compounds.

In a multiple metabolic network analysis, 19 strains of three Category A biothreat agents *Y. pestis, B. anthracis* and *F. tularensis* were examined toward having a common drug target. Nine essential enzymes were shared between these three high threat pathogens (Ahn et al., 2014). These enzymes are phosphopantothenoyl cysteine decarboxylase (CoaB), phosphopantothenate cysteine ligase (CoaC), pantetheine-phosphate adenylyltransferase (CoaD), dephospho-CoA kinase (CoaE) in coenzyme A biosynthesis pathway, dihydroneopterin aldolase (FolB), dihydrofolate synthase/tetrahydrofolate synthase (FolC, two distinct enzymatic activities), GTP cyclohydrolase I (FolE) in folate biosynthesis pathway, guanylate kinase (Gmk) and thymidylate kinase (Tmk) in nucleic acid pathways and phosphatidyl serine decarboxylase (Psd) in phosphatidyl-ethanolamine synthesis. The comparison of the predicted essential enzymes in common with the experimental studies showed that CoaE is essential for growth in *M. tuberculosis, S. aureus, H. influenza*, and four other bacteria. Gmk and Tmk were found to be essential in *B. subtilis, E. coli, H. influenza, S. aureus*, and *Mycoplasma genitalium*. A further study of whether existing antibiotics targeted to any of these nine enzymes demonstrated that the antimicrobial compounds, trimethoprim and Rab1, already targeted FolC. Trimethoprim directly inhibits dihydrofolate synthase activity and the accumulation of dihydrofolate indirectly inhibits tetrahydrofolate synthase in *E. coli* (Kwon et al., 2008). It is effective against *Y. pestis*, whereas *B. anthracis* is resistant against trimethoprim (Wong et al., 2000; Barrow et al., 2007). Rab1 demonstrated broad-spectrum applicability and inhibited growth of three category A biothreat agents besides methicillin-resistant *S. aureus* and vancomycin-resistant *S. aureus* (Bourne et al., 2010).

## Pathogen-Host Modeling

**Figure 3:**
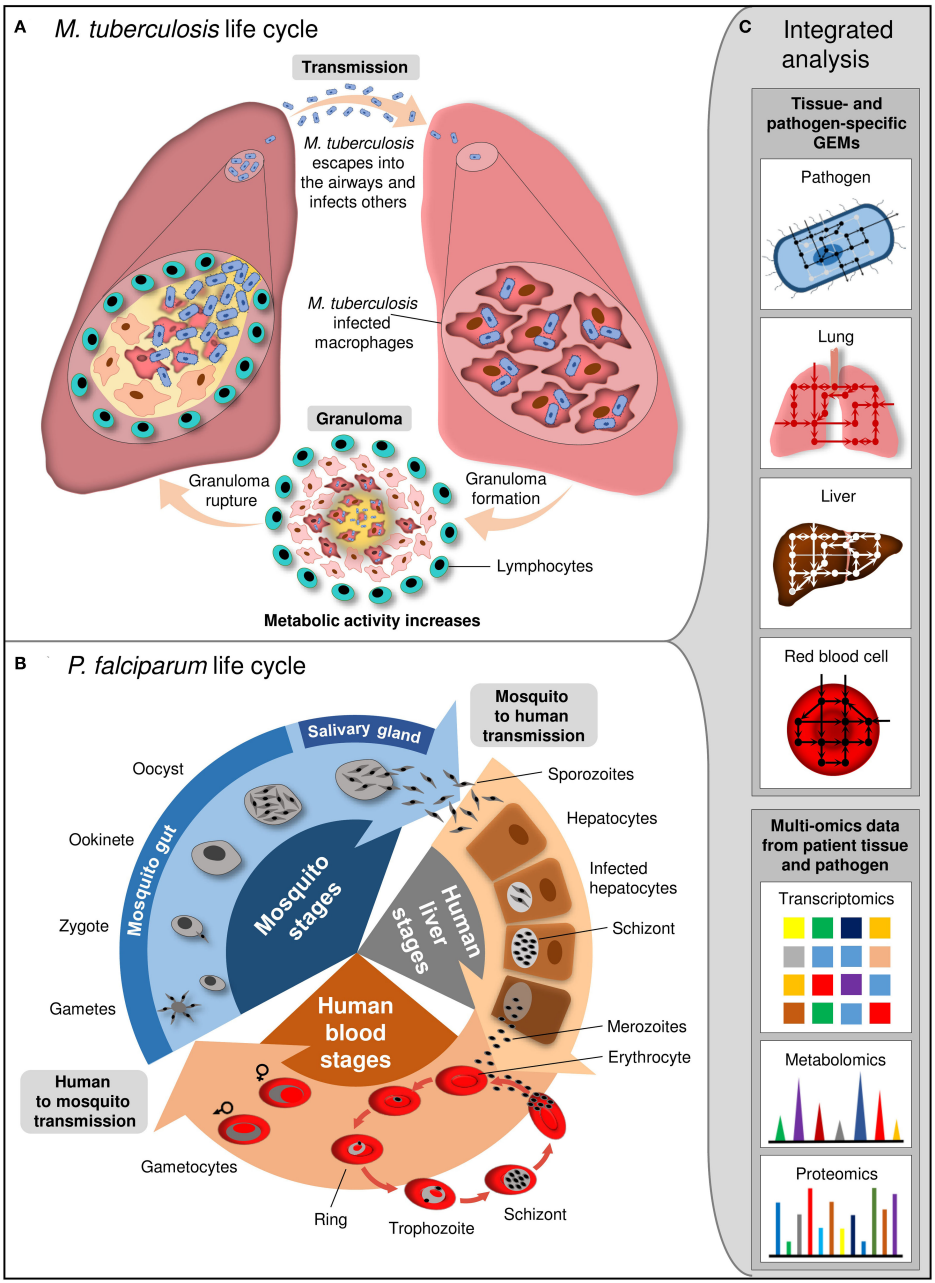
Life cycle of *M. tuberculosis* and *P. falciparum* and integrated analysis at system level. **(A)** *M. tuberculosis* is transmitted by aerosol. Inhaled pathogen reaches the alveoli of the lung and grows inside the alveolar macrophages. Granuloma, where *M. tuberculosis* kills the macrophages and escapes from the cell for division, is formed. Subsequent to maturation, granuloma ruptures and releases *M. tuberculosis* into the airways. **(B)** *P. falciparum* life cycle involves the different stages in female *Anopheles* mosquito and human. Mosquito transmits sporozoites into the human. They infect hepatocytes and mature into schizonts which release merozoites. Merozoites invade erythrocytes and resulted in release of newly multiplied merozoites by erythrocytes destruction. Some merozoites differentiate into gametocytes which are taken up from host by mosquito. Gametocytes develop into sporozoites within mosquito. **(C)** Integrated analysis of tissue- and pathogen-specific GEMs with the high-throughput multi-omics data provides insight into the cellular and interaction mechanisms between the pathogen and host tissue at different stage of infection.

Genome-scale metabolic models provide an improved understanding of how intracellular pathogens utilize the existing microenvironment of the host (Figure 3). It is of utmost importance to understand the pathogen metabolism including metabolic virulence factors like quorum sensing QS, lipopolysaccharides LPS, and rhamnolipids (and more such as siderophores-based iron uptake systems, cable pili for adhesion, motility, hemolysin, proteases, phospholipases, secretion systems, toxins, and extracellular capsules) to unravel mechanisms of pathogenesis. Pathogens reside in a phagosome (a vacuole in the cytoplasm of a cell), or more specifically localize in intracellular, extracellular-interstitial, extracellular-intravascular, extracellular-transcellular and “semi-open” spaces in host cells (e.g., the respiratory or alimentary tracts, etc.). Pathogens demonstrate different biochemical phenotypes and interaction mechanisms when inside the host vs. outside the host. Therefore, the pathogen and host GEMs are re-compartmentalized, and the transport across the macrophage cytoplasm are represented in connection with the other GEM.

An integrated host-pathogen GEM (iAB-AMØ-1410-Mt-661) was reconstructed by combining iAB-AMØ-1410 and iNJ661 which represent *in silico* alveolar macrophage and *M. tuberculosis* metabolism, respectively (Bordbar et al., 2010). iAB-AMØ-1410 was developed by using context-specific model extraction algorithms (GIMME and iMAT) and manual curation based on generic human GEM (Recon1) along with the gene expression data (Duarte et al., 2007; Becker and Palsson, 2008; Shlomi et al., 2008). The distribution of flux states for each reaction in the integrated model was compared with those of the corresponding reactions in the two pioneering models. In spite of the some variations in alveolar macrophage part, most of the changes were found in the pathogen part of the network where flux of glycolysis was reduced with acetyl-CoA synthesis produced from fatty acids. Glucose was produced through gluconeogenesis. Fatty acid oxidation pathways were upregulated; whereas, nucleotides, peptidoglycans and phenolic glycolipid productions were downregulated. In the alveolar macrophage part, nitric oxide production was increased; however, ATP synthesis, nucleotide production and amino acid metabolism were reduced. Due to different infection mechanisms in different tissues, three infection-specific models were developed for latent, pulmonary and meningeal tuberculosis by mapping gene expression data (Thuong et al., 2008). Hyaluronan synthesis was computed to be active only in the pulmonary-specific model, suggesting that the inhibition of hyaluronan synthase as a putative approach to cease the activation from latent to pulmonary condition. Furthermore, vitamin D and folate metabolism were found to be active in pulmonary and meningeal states. Vitamin D is important to fight against infection and folate play a critical role in DNA synthesis and repair.

*Salmonella* metabolism during infection was studied by using host-cell nutrient environment and gene expression data obtained from *S. typhimurium* growing inside macrophage cell lines (Raghunathan et al., 2009). The genome-scale metabolic model (iRR1083) was reconstructed specific to *S. typhimurium* LT2. FBA and FVA were employed in the prediction of essential genes and active metabolic pathways during infection. 417 and 736 flux-carrying reactions were computed for optimal and suboptimal biomass productions, respectively. Gene expressions and nutrients availability in the host cell environment were used as constraints in the simulations. Blocked reactions were predicted to offer valuable insight into inactive pathways in *Salmonella* metabolism under investigated conditions during infection. Integrated gene expression and optimal growth computations resulted in unexpressed *fad* genes in fatty acid degradation; however, the same genes were predicted to be operational under suboptimal growth conditions. Therefore, the simulations of *in silico* iRR1083 model provided clues toward metabolic shifts from early stages of infection to chronic infection of *Salmonella*. The computational results were compared with the literature-based proteome data, where 315 *S. typhimurium* proteins were identified by isolating this pathogen from macrophage at different time following infection (Shi et al., 2006). Of the 129 proteins, which were shared between experimental proteome data and iRR1080 model, 80 and 34 proteins were found within the reactions computed by FVA during optimal and suboptimal growth, respectively.

Prediction of essential genes for enterobacterial human pathogens (three *E. coli* and one *Salmonella* strains) was performed in three different host niches including human bloodstream, urinary tract and macrophage (Ding et al., 2016). Together with *E.coli* pangenome metabolic model (iEco1712_pan) containing all metabolic reactions and associated genes from 16 *E. coli* genome, the experimentally-based nutrient compositions were used as simulation constraints to mimic these three host environments (Keiter et al., 1955; Putnam, 1971; Raghunathan et al., 2009; Baumler et al., 2011). Among 51 metabolites, only 15 of them were shared for all niches of interest since the different locations inside the human body have diverse nutrients availability, which plays a pivotal role in pathogen survival and infection mechanism. By deleting each gene, essential (no growth) and important (reduction of biomass production >1% of the wild type) genes were predicted through FBA. Only one important reaction (isocitrate dehydrogenase) and 38 essential reactions including allantoinase, ATP synthase, citrate lyase, enolase and pyruvate kinase were predicted in common for all host locations. However, 121 reactions were computed to be important in one or two host environment. Subsequent to defining essential and important genes, these genes were compared with genomes of *E. coli* UT189, *E. coli* 53638, and *Salmonella* LT2 to investigate whether these genes were retained or lost over time. The genome of *E. coli* O157:H7 was utilized as control since it causes infection in a different location of human body (intestine). Therefore, this control strain lost highest number of these genes predicted for each of the three host environments. As expected, most of the lost essential or important genes were computed in intestinal pathogen *E. coli* O157:H7 due to evolutionary outcomes. The simulations of *E. coli* UT189 successfully predicted least amount of essential and important genes lost in human bloodstream and urinary tract where it causes infection. However, the inconsistencies in urinary tract simulations demonstrated a necessity of additional constrains for more accurate predictions in this location.

Similar to host-pathogen modeling at system-level, unraveling of host-parasite interactions via genome-scale metabolic modeling draws researchers' attention. A good example in this sense is the parasite *Plasmodium falciparum* which is responsible for the most severe form of malaria and involves the different stages in mosquito and human (Figure 3). Subsequent to reconstructions and integration of GEM for *P. falciparum* and human erythrocyte, antimalarial drug targets were predicted for stage-specific conditions by mapping gene expression data from different life cycles (Huthmacher et al., 2010). Among 57 experimentally supported essential enzymes, 35 enzymes including glutathione reductase, thioredoxin reductase, carbonic anhydrase and acetyl-CoA carboxylase were predicted to be putative drug targets. By applying additional assumptions (transporter constraints of biomass precursors), another 16 enzymes including spermidine synthase and ornithine decarboxylase were also predicted to have antimalarial effects. Drug targets of this parasite in human liver metabolism were simulated by using well-curated GEMs for human hepatocyte (HepatoNet1) and *P. falciparum* (PlasmoNet) (Gille et al., 2010; Huthmacher et al., 2010; Bazzani et al., 2012). Twenty-four out of 48 experimental antimalarial drug targets including serine transferase, sphingomyelin synthase, thioredoxin reductase, and adenylosuccinate synthase were predicted as essential for parasite and non-essential for human. Another study was carried out to elucidate host response to malarial infection during the intraerythrocytic developmental cycle (IDC) (Wallqvist et al., 2016). Along with time series gene expression data during IDC, the proteomic based reconstruction of red blood cell (iAB-RBC-283) and metabolic network model of *P. falciparum* were coupled to simulate infection metabolism of this parasite inside the human red blood cells (Bordbar et al., 2011; Fang et al., 2014). The glycolytic pathway fluxes of co-cultured uninfected red blood cells were predicted between 13 and 19% of those in normal cells, indicating inactivation of glycolysis pathway due to the presence of infected cells in the same culture. Temporal activation of the glycolytic pathway was observed among the infected and co-cultured red blood cells. During very early and late intraerythrocytic developmental cycle (IDC), infected and co-cultured red blood cells showed similar patterns with high fluxes through phosphoglycerate kinase and low fluxes through diphosphoglycero mutase and diphosphoglycerate phosphatase. However, the low fluxes through phosphoglycerate kinase were predicted in the infected red blood cells during the middle IDC because of ATP secretion from *P. falciparum*.

Considering the different life cycle stages (liver, blood, and mosquito stage of parasites), the metabolism of the parasite *P. falciparum* varies due to fluctuating availability of nutrients in the different host environments. Thus, a stage-specific metabolism of *P. falciparum* was studied by a context specific GEM using gene expression data to get an idea during which stages reactions are essential (Huthmacher et al., 2010). The *in silico* analysis predicted 307 essential reactions for the parasite. All reactions were computed as essential during at least one developmental stage. In blood stages of its life cycle, the parasite *P. falciparum* need to incorporate heme from hemoglobin degradation into hemozoin molecules in order to prevent intoxication and cell lysis. Consistent with this, the predictions demonstrated several essential enzymes involved in heme biosynthesis. In late liver stage, the fatty acid synthesis is only essential in the apicoplast. Essential reaction predictions demonstrated that top ranking reactions were found in the apicoplast. In ring stage, fewer reactions corresponding to folate biosynthesis are predicted to be active compared to subsequent stages. In schizont and early ring stage, an increase in the number of active reactions is predicted for the citric acid cycle and sphingolipid metabolism. In trophozoite and schizont stages, the parasites have a high acyltransferase activity, that can be considered as significant for their fitness (Huthmacher et al., 2010; Bazzani et al., 2012). Among the metabolic reactions, *P. falciparum* involves a pathway to synthesize pyrimidine nucleotides *de novo* of which multiple reactions were predicted to be essential such as those catalyzed by carbonic anhydrase, carbamoylphosphate synthase, dihydroorotase, dihydroorotate oxidase, orotidine-5′-phosphate decarboxylase. Moreover, the enzymatic inhibitions of *Plasmodium falciparum* were investigated by a reconstructed genome-scale network PlasmoNet (Bazzani et al., 2012). Acyl-CoA synthetase inhibition impaired PlasmoNet sphingomyelin production, suggesting a certain amount of sphingolipids is essential for the growth of *Plasmodium falciparum*. Similarly, aspartate carbamoyltransferase inhibition resulted in an impairment of UDP-glucose production in PlasmoNet. The *in silico* inhibition of the enzyme glycerol-3-phosphate acyltransferase impaired the production of phosphatidylethanolamine in PlasmoNet. This enzyme glycerol-3-phosphate acyltransferase is essential for *P. falciparum* growth that necessitates high amount of phospholipids for membrane synthesis.

Several studies have indicated that the time-of-day of host infection influences pathogen progression (Rijo-Ferreira and Takahashi, 2019). For example, the levels of *Salmonella enterica subsp. enterica* serovar Typhimurium (*S. typhimurium*) were higher if the infection occurred during the rest phase compared to the infection initiated in the middle of the active phase (Bellet et al., 2013). Viral infections of herpes, influenza A, and respiratory viruses of the Paramyxoviridae family were worse when host circadian rhythms are disrupted, e.g., by mutation in *Bmal1* gene, the main circadian regulator. The parasite infection similarly depends on the timing of the host circadian cycle, e.g., the load of *Leishmania* parasite is circadian in nature. The circadian clock (mainly BMAL1) can regulate cellular immunity against bacteria, viruses, and parasites (Rijo-Ferreira and Takahashi, 2019). Infections along with the resulting inflammation can disrupt the circadian clock by decreasing the amplitude of circadian rhythms. Some examples are seen with *Trypanosoma cruzi, Trypanosoma brucei, Plasmodium chabaudi* (Fernández Alfonso et al., 2003; Rijo-Ferreira et al., 2018).

Host-pathogen (HP) models have unraveled the pathogen adaptation and carbon source utilization *in vivo* and host manipulation by pathogen. These HP models answer questions regarding the causality during the infection process, condition dependent (or context specific) differences. The diagnosis and treatment related challenges can be solved by examining the metabolic fluxes in tissues and develop strategies for treatment options on the basis of few experimental data. In brief, HP models help elucidate the role of host environment on pathogen metabolism during the course of an infection.

## Sphingolipids Related Invasion Mechanism of Pathogens

During the microbial infection process, the sphingolipid molecules play a critical role in host-pathogen interaction mechanism (Sharma and Prakash, 2017; Kunz and Kozjak-Pavlovic, 2019; Rolando and Buchrieser, 2019). The initial steps of infection is the host-pathogen contact on the cell surface and then penetration of pathogen into the host cell. Many of the pathogens do not have their own sphingolipids; however, they are able to take advantage of host sphingolipid pathway to promote their virulence and invasion. Sphingolipids are bioactive lipids participating in cell membrane and various cellular processes including growth, death, adhesion, inflammation and signaling (Hannun and Obeid, 2008). Ceramide is the central hub metabolite in sphingolipid metabolism due to different formation (*de novo* synthesis from palmitoyl-CoA and serine, sphingomyelin hydrolysis by sphingomyelinases and reacylation of sphingosine catalyzed by ceramide synthase) and consumption reactions covering conversion to sphingosine, sphingomyelin, glucosylceramide and ceramide 1-phosphate catalyzed by the enzymes of ceramidase, sphingomyelin synthase, glucosylceramide synthase and ceramide kinase, respectively.

Acid sphingomyelinase (ASM) converts sphingomyelin to ceramide and has a great importance in membrane reorganization. Several bacterial pathogens activate the ASM and ceramide-enriched membrane platforms are formed in response to increase in ceramides (Simonis and Schubert-Unkmeir, 2018). These lipid rafts facilitate the uptake of bacterial pathogen into host. Moreover, different pathogens demonstrate specific infection mechanisms based on their own and host cell characteristics. In *P. aeruginosa* infection in cystic fibrosis, the increase in ceramide and ceramide-enriched platforms, where β1-integrins are located on the luminal pole of bronchial cells, lead to the accumulation of β1-integrins (Grassmé et al., 2017). The downregulation of acid ceramidase expression due to β1-integrins causes further accumulation of ceramide and a decrease in surface sphingosine, which kills bacteria. *S. aureus* α-toxin activates ASM and concomitant formation of ceramide in endothelial cells by means of binding to ADAM10 (Becker et al., 2018; Keitsch et al., 2018). In addition to ceramide, sphingosine and sphingosine-1-phosphate (S1P) participate in lung inflammatory injury. S1P is produced by the sphingosine kinase-1 (SPHK1) in cytosol and sphingosine kinase-2 (SPHK2) in nucleus by phosphorylation of sphingosine. *P. aeruginosa* infection resulted in phosphorylation of SPHK2 and increased localization in nucleus leading to enhanced level of S1P and acetylation of histone (Ebenezer et al., 2019). *M. tuberculosis*, which is directly able to use glycosphingolipids of the plasma membrane, can bind to lactosylceramide-enriched lipid rafts of human neutrophils (Nakayama et al., 2016). Sphingomyelin is necessary for *H. pylori* entry, and secreted vacuolating cytotoxin facilitates bacterial colonization (Foegeding et al., 2016).

The successful response of host cell to bacterial invasion takes full advantages of phagolysosome formation or autophagy induction to kill bacteria. On the other hand, pathogens develop various strategies to survive including escaping from phagosome into the cytosol, inhibiting phagocytosis, preventing fusion of phagosomes and lysosomes and blocking autophagy. Sphingolipids participate in these strategies for pathogenic survival in the host. The protein Rv0888 which shows sphingomyelinase activity is synthesized by *M. tuberculosis*. This protein converts host sphingomyelin into ceramide and phosphorylcholine, and these metabolites are used as carbon, nitrogen and phosphorus sources by *M. tuberculosis* (Speer et al., 2015). *P. aeruginosa* infection activates the mitochondrial ASM (Managò et al., 2015). This results in cell death due to formation of mitochondrial ceramide and the release of mitochondrial cytochrome c. Subsequent to invasion, a type-III secretion system is required for *Salmonella* to constitute a specialized bacterial survival and replication niche, which is called *Salmonella*-containing vacuole (SCV) (Owen et al., 2014). Based on the replication stage, *Salmonella* can induce suppression of autophagy so as to increase survival. This can be regulated by sphingolipids since the decreased *Salmonella*-induced autophagy results from inhibition of *de novo* sphingolipid biosynthesis (Huang, 2016).

These examples show that pathogens are willing to use and manipulate sphingolipids in order to invade eukaryotic cell and promote pathogen colonization in the host. The cases are not limited to the above mentioned examples, and can be extended to further pathogens like *Neisseria* strains (*Neisseria meningitidis, Neisseria gonorrhoeae*), *H. influenzae, Chlamydia trachomatis, Legionella pneumophila, Candida albicans, Cryptococcus neoformans, Aspergillus fumigatus*, etc. (Aerts et al., 2019; Kunz and Kozjak-Pavlovic, 2019; Rolando and Buchrieser, 2019). Pharmaceutical reduction of sphingolipids and glycosphingolipids such as glucosylceramide (GlcCer) was proposed as a new strategy to fight against fungal infections. Acylhydrazones have been determined as specific inhibitors since they targets synthesis of fungal and not mammalian GlcCer (Mor et al., 2015; Lazzarini et al., 2018).

## Concluding Remarks

Pathogen-specific genome-scale metabolic models as well as host-pathogen integrative constraint-based methods have become successful tools in the research of infection mechanism that need to be fully understood to develop therapeutical strategies against pathogens. The analysis of essential genes and their associated products provide a valuable insight into how to impair bacterial growth and how these critical biomolecules change in different environmental states which mimic different human niches. Essential biomolecules common in different infected host microenvironment have high potential for targeting in the combat of pathogen in multiple human niches. Similarly, the different stages of infection may show stage-specific essential biomolecules to be targeted by antimicrobial agents. In such cases, molecules shared in all stages are selected as putative drug targets. The identification of conserved metabolic pathways during pathogen invasion may lead to alternative complementary routes that can be targeted by novel interfering compounds. If it is not possible to treat the bacterial infection with a single antibiotic, combined treatment strategies (cocktail of drugs) would be an alternative solution. For the rare bacterial species involved in infections, an economically feasible route for pharmaceutical companies may be the search for broad spectrum antibiotics. Another option would be the development of combinatorial treatment protocols using current drugs that are already in use against infections. Here, it is strictly necessary not to forget the human orthologs, which need to be excluded from being drug targets in order to avoid side effects. The computational genome scale models presented in this review article have not only the ability to reduce the search space for novel drug targets in the pathogen metabolism, but can also give insights on putative side effects on host metabolism. These metabolic network models can be used as an additional screening tool to predict potential toxicity of the predicted target in a healthy cell or tissue model. Many side effects are due to patient-to-patient genetic variability. Using context specific constraint-based models integrated with omics data, the potential causes of the side effects such as drug off-target binding, downstream transcriptional effects (changes in gene expression that are induced with drug treatment), and the pharmacokinetics of drug clearance can be predicted in patients and serve as *a priori* guide in the drug target studies.

In case of extremely deadly pathogens, this genome scale modeling approach is indispensable since experimental analyses are associated with the need for high-security laboratory conditions, and in some cases prohibited. These models identify novel virulence genes as well as enable the evaluation of pathogen metabolism, predict disease phenotypes and hence get the infection scenario. They offer insight into why the same microbial pathogen may cause disease in some host environments but not others.

Pathogen species, infection stages and host niches have great importance and need to be taken into consideration in clinical and computational studies to extend curative strategies against pathogens for global preventative healthcare. Throughout the infection course, different pathogens develop various stage-specific interaction mechanisms to host cells depending on their invasion and response features. The experimental elucidation of such complex infection process requires long and difficult work alone. An effective application of stage-specific genome-scale metabolic modeling approach guides experimental studies and facilitate the development of novel candidate drugs targeting key essential biomolecules at all stages infection.

## Author Contributions

MS and KOU contributed to writing the manuscript and preparing the table and figures. Both authors contributed to the article and approved the submitted version.

## Conflict of Interest

The authors declare that the research was conducted in the absence of any commercial or financial relationships that could be construed as a potential conflict of interest.
